# Association of Serum 25(OH)D, Cadmium, CRP With All-Cause, Cause-Specific Mortality: A Prospective Cohort Study

**DOI:** 10.3389/fnut.2022.803985

**Published:** 2022-04-27

**Authors:** Yan Liu, Donghui Yang, Fang Shi, Fang Wang, Xiaoxue Liu, Haoyu Wen, Sumaira Mubarik, Chuanhua Yu

**Affiliations:** ^1^School of Public Health, Wuhan University, Wuhan, China; ^2^School of Public Health, Xuzhou Medical University, Xuzhou, China

**Keywords:** serum 25(OH)D, cadmium, C-reactive protein, mortality, diabetic

## Abstract

**Introduction:**

To explore the relationship between serum 25(OH)D, cadmium, and CRP with all-cause mortality among people in diabetic and non-diabetic.

**Methods:**

This study used data from the NHANES (2001–2010). Cox regression was used to analyze the relationship between Serum 25(OH)D, cadmium, CRP, and all-cause, cause-specific mortality. We used restricted cubic splines to explore the dose-response relationship between serum 25(OH)D, cadmium, CRP, and all-cause mortality.

**Results:**

During a mean follow-up of 9.1 years, the study included 20,221 participants, 2,945 people with diabetes, and 17,276 people without diabetes. Compared with serum 25(OH)D deficiency group in diabetic patients, the sufficient serum 25(OH)D group was associated with lower all-cause mortality (HR = 0.41, 95%CI 0.28-0.60, *P* < 0.001) and cardiovascular mortality (HR = 0.46, 95%CI 0.22–0.95, *P* = 0.04). Compared with the low cadmium group, the high cadmium group was associated with higher all-cause mortality (HR = 1.49, 95%CI 1.06–2.09, *P* = 0.02). Compared with the low CRP group, the high CRP group was associated with higher all-cause mortality (HR = 1.65, 95%CI 1.24–2.19, *P* = 0.001) and cancer mortality (HR = 3.25, 95%CI 1.82–5.80, *P* < 0.001). Restricted cubic splines analysis showed a significant nonlinear association between serum 25(OH)D (*P*-nonlinearity *P* < 0.001), cadmium (*P*-nonlinearity = 0.002), CRP (*P*-nonlinearity = 0.003), and HR for all-cause mortality risk in diabetic patients. The results were similar among non-diabetic patients, but with different levels of risk. Sensitivity analysis and subgroup analysis presented the results of population studies with different follow-up times, different genders and ages.

**Conclusions:**

In diabetic patients, serum 25(OH)D, cadmium, and CRP were related to all-cause mortality; serum 25(OH)D was related to cardiovascular mortality; CRP was related to cancer mortality. The results were similar among non-diabetic patients, but with different levels of risk.

## Introduction

International Diabetes Federation (IDF) estimated the global diabetes prevalence in 2019 was estimated to be 9.3% (463 million people), it is expected to reach 10.9% (700 million) by 2045 (1). Cardiovascular disease (CVD) was the main cause of death and morbidity in patients with diabetes, especially type 2 diabetes (2). Therefore, it was of great significance to explore the causes of diabetes, prevent or control the complications of diabetes and reduce the risk of death.

Vitamin D was believed to prevent common chronic diseases, including CVD and cancer (3, 4). In a meta-analysis of randomized controlled trials, vitamin D supplementation significantly reduced overall cancer mortality (5). However, clinical data examining the effect of vitamin D supplementation on mortality was still inconclusive (6, 7). The relationship between vitamin D status and the risk of death in diabetic patients was not consistent (8, 9). This may be related to the different sample size, the definition of vitamin D deficiency, and the different adjustment of covariates in the detection method. There was increasing interest in the possible protective effects of vitamin D in health outcomes.

Cadmium (Cd) is a toxic heavy metal that causes various diseases and increases mortality. Diet is the main source of Cd exposure for most people. A survey from NHANES in 2007–2012 showed that the food groups with the most Cd were grains and breads, leafy vegetables, potatoes, beans and nuts, and stem/root vegetables (10). The cadmium content in food is related to geographic location, bioavailability of cadmium in soil, crop genetics and agronomy (11). Cadmium exposure was associated with kidney disease (12), osteoporosis and fractures (13), atherosclerosis (14) and CVD (15). The relationship between epidemiological studies of low-level cadmium exposure and the risk of cancer and cardiovascular disease was limited, and there are usually fewer cohort studies based on small sample sizes, case-control, or cross-sectional designs. A meta-analysis showed that cadmium appears to be associated with increased all-cause mortality and cardiovascular mortality even at low levels of exposure (16). But this meta-analysis contains only 9 original studies, and the heterogeneity was great. In addition, we also separated the diabetic and non-diabetic for analyses.

C-reactive protein (CRP) was a protein synthesized in the liver, and the inflammatory response of tissue damage was related to the level of CRP (17). Despite the large number of studies on CRP, the convincing evidence on associations and causal effects was very limited (18). A study using the China Longitudinal Study of Health and Retirement (CHARLS) showed that plasma CRP was a predictor of all-cause mortality in the middle-aged and elderly population in China (19). A cohort study in Brazil also reached the same conclusion (20). Meta-analysis showed that CRP levels were associated with all-cause mortality in patients with type 2 diabetes and the risk of cardiovascular mortality was higher (21). Our data with larger samples and longer follow-up time. In addition, we also separated the diabetic and non-diabetic for analyses.

This research attempted to explore the relationship between serum 25(OH)D, cadmium, and CRP with all-cause mortality among people in diabetic and non-diabetic.

## Methods

### Study Population

This study used data from the NHANES (2001–2010). The National Health and Nutrition Examination Survey (NHANES) is a program of studies designed to assess the health and nutritional status of adults and children in the United States. NHANES is a major program of the National Center for Health Statistics (NCHS). NCHS is part of the Centers for Disease Control and Prevention (CDC) and has the responsibility for producing vital and health statistics for the Nation. The survey examines a nationally representative sample of about 5,000 persons each year. These persons were located in counties across the country, 15 of which were visited each year. The NHANES interview includes demographic, socioeconomic, dietary, and health-related questions. The examination component consists of medical, dental, and physiological measurements, as well as laboratory tests administered by highly trained medical personnel.

We collated and merged the data from 2001 to 2010 and obtained 52,195 respondents. After the deletion of missing research variables (serum vitamin D, blood cadmium, C-reactive protein) and covariates (age, gender, education, marital status, race, ratio of family income to poverty, BMI, drinking, smoking, physical activity), 20,221 respondents were finally obtained. In the end, there were 2,945 diabetic people and 17,276 non-diabetic people. Diabetes is defined as self-reported as diabetes when asked by a doctor, using insulin or oral hypoglycemic drugs, fasting blood glucose ≥7.0 mmol/L, or glycosylated hemoglobin A1c (HbA1c) ≥6.5%. Gestational diabetes is excluded.

### Measurement of Serum 25(OH)D

NHANES 2001–2006, serum 25(OH)D concentration was measured by DiaSorin radioimmunoassay kit (Stillwater, MN), but the data was converted to equivalent 25(OH)D measured value from LC-MS/MS method by regression method. From 2007 to 2010, serum 25(OH)D concentration was measured by a standardized liquid chromatography-tandem mass spectrometry (LC-MS/MS) method. The CDC LC-MS/MS method has better analytical specificity and sensitivity compared to immunoassay methods, and fixed analytical goals for imprecision ( ≤ 10%) and bias ( ≤ 5%).

### Measurement of Serum Cadmium

We used data of blood cadmium. Whole blood samples were processed, stored under frozen (−30°C) conditions, and shipped to the Ministry of Laboratory Science, the National Center for Environmental Health, and the Centers for Disease Control and Prevention for analysis. Inductively coupled plasma mass spectrometry (ICP-MS) was used to determine the concentration of cadmium (Cd) in whole blood.

### Measurement of Serum C-Reactive Protein

C-reactive protein was considered to be one of the best measures of acute-phase response to infectious diseases or other tissue damage and inflammation. The blood samples are processed, stored, and shipped to the University of Washington. Quantify CRP by latex-enhanced turbidimetry. The CRP concentration is calculated by using a calibration curve. Data reduction of the signals was performed by using a storable logit-log function for the calibration curve performed data reduction of the signals. These measurements were performed on the Behring Nephelometer for quantitative CRP measurement.

### Ascertainment of Mortality

NCHS has linked various surveys with death certificate records from the National Death Index (NDI). The restricted-use Linked Mortality File (LMF) has been updated with mortality follow-up data through December 31, 2015. All-cause mortality, cardiovascular disease, and cancer mortality were determined by correlation with the National Death Index. ICD-10 is used to determine deaths from specific diseases. Cancer mortality was defined as ICD-10 codes C00–C97, and CVD mortality was defined as ICD-10 codes I00–I09, I11, I13, I20–I51, or I60–I69.

### Assessment of Covariates

The covariates we included age, gender, education, marital status, race, the ratio of family income to poverty, BMI, drinking, smoking, physical activity. Education level was categorized as less than high school, more than high school. Marital status was categorized as married or living with a partner; widowed, divorced, separated; never married. The race was classified as Hispanic, non-Hispanic-White, non-Hispanic- Black, non-Hispanic other. The family income-to-poverty ratio was classified as 0–1.0, 1.0–3.0, and >3.0. BMI was categorized as <25.0 kg/m^2^, 25.0–29.9 kg/m^2^, and ≥30 kg/m^2^. Drinking status was categorized as never drinker (less than 12 alcohol drinks/lifetime), Ever drinker (had at least 12 alcohol drinks/1 yr and no drink alcohol over past 12 months), current drinker (drink alcohol over past 12 months). Smoking was categorized as never smoker (smoked less than 100 cigarettes in life), ever smoker (smoked at least 100 cigarettes in life and no smoke now), current smoker (smoked at least 100 cigarettes in life and smoke now). Physical activity was categorized as <150 min MVPA(moderate to vigorous physical activity), ≥150 min MVPA 0. 75 min of vigorous physical activity is equal to 150 min of moderate physical activity (22). In addition, strict procedures were applied for collection and analysis in the whole blood, and the details were described in the NHANES laboratory.

### Statistical Analysis Method

This study describes the basic situation of objects. We generated a new weight for 2001–2010 = wtmec2yr/5. Cox regression analysis was used to analyze the relationship between serum 25(OH)D, cadmium, CRP, and all-cause and cause-specific mortality. According to the clinical practice guidelines of the Endocrine Society, serum 25(OH)D status is divided into four groups: severe deficiency (<25.0 nmol/L), moderate deficiency (25.0–49.9 nmol/L), insufficient (50.0–74.9 nmol/L), and sufficient (>75.0 nmol/L) (23). Cadmium and CRP were also divided into four groups according to the interquartile range. We conducted a subgroup analysis based on age and gender. At the same time, we conducted sensitivity analyses by excluding subjects whose follow-up time was less than 2 years.

We also used restricted cubic splines (RCS) with three knots (5th, 50th, and 75th) to explore the dose-response relationship between serum 25(OH)D, cadmium, CRP, and all-cause mortality. At the same time, we adjusted multiple covariates mentioned above. The essence of restrictive cubic splines is regression splines with additional requirements. The regression spline is a piecewise polynomial that requires continuous and second-order derivable in each piece. Restricted cubic splines require regression splines: the spline function was a linear function at the extreme ends of the independent variable data range.

All of the analyses were conducted using Stata 15.0. *P* > 0.05 was considered statistically significant.

## Results

### Baseline Characteristics

The study included 20,221 participants, 2,945 people with diabetes, and 17,276 people without diabetes. The average age of the diabetic population is 61.6 years old, and the average age of the non-diabetic population is 47.4 years old. The specific baseline characteristics of the study population were shown in Table 1. People with diabetes were more likely to be older, non-Hispanic black, low-educated, obese people who currently drink alcohol and do not exercise enough.

**Table 1:** Baseline characteristics of participants in the NHANES 2001–2010.

**Baseline characteristics**	**Total**	**Diabetes**		**Non-diabetes**	
		**Male**	**Female**	**Male**	**Female**
Sample size (%)	20,221	1,565 (7.7)	1,380 (6.8)	8,330(41.2)	8,946(44.2)
**Age (years) (%)**					
<65	15,189	843 (5.6)	747 (4.9)	6,467 (42.6)	7,132 (47.0)
≥65	5,032	722 (14.3)	633 (12.6)	1,863 (37.0)	1,814 (36.0)
**Race (%)**					
Hispanic	5,095	454 (8.9)	430 (8.4)	2,009 (39.4)	2,202 (43.2)
Non-hispanic white	10,597	724 (6.8)	536 (5.1)	4,498 (42.4)	4,839 (45.7)
Non-hispanic black	3,759	341 (9.1)	357 (9.5)	1,503 (40.0)	1,558 (41.4)
Non-hispanic other	770	46 (6.0)	57 (7.4)	320 (41.6)	347 (45.1)
**Education level (%)**					
Less than high school degree	5,636	616 (10.9)	577 (10.2)	2,261 (40.1)	2,182 (38.7)
High school degree	4,739	334 (7.0)	334 (7.0)	2,064 (43.6)	2,007 (42.4)
More than high school degree	9,776	615 (6.3)	469 (4.8)	4,005 (41.0)	4,687 (47.9)
**Marital status (%)**					
Married or living with partner	12,678	1,116 (8.8)	664 (5.2)	5,652 (44.6)	5,246 (41.4)
Widowed, divorced, separated	4,414	333 (7.5)	619 (14.0)	1,181 (26.8)	2,281 (51.7)
Never married	3,129	116 (3.7)	97 (3.1)	1,497 (47.8)	1,419 (45.3)
**Family income-poverty ratio (%)**					
0–1.0	3,792	270 (7.1)	354 (9.3)	1,430 (37.7)	1,738 (45.8)
1.1–3.0	8,478	727 (8.6)	693 (8.2)	3,378 (39.8)	3,680 (43.4)
>3.0	7,951	568 (7.1)	333 (4.2)	3,522 (44.3)	3,528 (44.4)
**BMI,kg/m** ^**2**^					
<25.0	5,989	228 (3.8)	174 (2.9)	2,487 (41.5)	3,100 (51.8)
25.0–29.9	7,097	532 (7.5)	353 (5.0)	3,454 (48.7)	2,758 (38.9)
≥30	7,135	805 (11.3)	853 (12.0)	2,389 (33.5)	3,088 (43.3)
**Drinking status (%)**					
Never drinker	2,792	129 (4.6)	418 (15.0)	568 (20.3)	1,677 (60.1)
Ever drinker	4,089	531 (13.0)	448 (11.0)	1,478 (36.1)	1,632 (39.9)
Current drinker	13,340	905 (6.8)	514 (3.9)	6,284 (47.1)	5,637 (42.3)
**Smoking Status (%)**					
Never smoker	10,445	567 (5.4)	809 (7.7)	3,630 (34.8)	5,439 (52.1)
Ever smoker	5,332	689 (12.9)	373 (7.0)	2,481 (46.5)	1,789 (33.6)
Current smoker	4,444	309 (7.0)	198 (4.5)	2,219 (49.9)	1,718 (38.7)
**Physical activity (%)**					
<150 min MVPA	11,375	990 (8.7)	1,051 (9.2)	3,958 (34.8)	5,376 (47.3)
≥150 min MVPA	8,846	575 (6.5)	329 (3.7)	4,372 (49.4)	3,570 (40.4)

*Values are n (percentage)*.

### All-Cause and Cause-Specific Mortality

In the follow-up of people with diabetes, 808 deaths were recorded, including 198 deaths from CVD and 154 deaths from cancer. In the Cox regression analysis, Model 1 adjusts age and gender; Model 2 further adjusted education, marital status, race, the ratio of family income to poverty based on model 1; Model 3 adjusted BMI, drinking, smoking, physical activity based on Model 2. We used the severe serum 25(OH)D deficiency group as the reference group, the sufficient serum 25(OH)D group was associated with lower all-cause mortality (HR = 0.41, 95%CI 0.28–0.60, *P* < 0.001) and cardiovascular mortality (HR = 0.46,95%CI 0.22–0.95, *P* = 0.04). We divided diabetic patients into 4 groups based on the interquartile range of cadmium concentration in the blood. At the same time, the low-concentration group (<0.24 μg/l) was used as the control group, the results showed that higher blood cadmium concentration group (>0.6 μg/l) was associated with higher all-cause mortality (HR = 1.49, 95%CI 1.06–2.09, *P* = 0.02). We divided diabetic patients into 4 groups based on the interquartile range of CRP concentration in the blood. At the same time, the low concentration group (<0.08 mg/dL) was used as the control group, the higher CRP concentration group (>0.49 mg/dL) was associated with higher all-cause mortality (HR 1.65, 95%CI 1.24–2.19, *P* = 0.001) and cancer mortality (HR = 3.25, 95%CI 1.82–5.80, *P* < 0.001). Other details were presented in Table 2.

**Table 2:** HR (95%CIs) for all-cause and cause-specific mortality according to serum 25 (OH)D, Cadmium and CRP among participants with diabetes.

**Life styles**	**Deaths**	**Total population**	**Model 1^a^**		**Model 2^b^**		**Model 3^c^**	
			**HR (95%CI)**	***P*-value**	**HR (95%CI)**	***P*-value**	**HR (95%CI)**	***P*–value**
**25 (OH)D**								
**All-cause mortality**								
<25.0 (nmol/L)	65	188	1.00 [Reference]		1.00 [Reference]		1.00 [Reference]	
25.0–49.9 (nmol/L)	302	1,118	0.56 (0.39- 0.80)	0.002	0.54 (0.37–0.78)	0.001	0.55 (0.39–0.79)	0.001
50.0–74.9 (nmol/L)	303	1,132	0.39 (0.28–0.54)	<0.001	0.36 (0.25–0.52)	<0.001	0.40 (0.27–0.58)	<0.001
>75.0 (nmol/L)	138	507	0.39 (0.28–0.55)	<0.001	0.38 (0.26–0.55)	<0.001	0.41 (0.28–0.60)	<0.001
**CVD mortality**								
<25.0 (nmol/L)	20	188	1.00 [Reference]		1.00 [Reference]		1.00 [Reference]	
25.0–49.9 (nmol/L)	68	1,118	0.47 (0.24–0.92)	0.03	0.46 (0.24–0.86)	0.02	0.45 (0.24–0.58)	0.01
50.0–74.9 (nmol/L)	69	1,132	0.29 (0.16–0.53)	<0.001	0.30 (0.17–0.51)	<0.001	0.31 (0.17–0.55)	<0.001
>75.0 (nmol/L)	41	507	0.44 (0.20–0.99)	0.048	0.46 (0.22–0.98)	0.04	0.46 (0.22–0.95)	0.04
**Cancer mortality**								
<25.0 (nmol/L)	6	188	1.00 [Reference]		1.00 [Reference]		1.00 [Reference]	
25.0–49.9 (nmol/L)	76	1,118	1.96 (0.74–5.20)	0.17	1.89 (0.70–5.07)	0.21	2.16 (0.78–5.95)	0.13
50.0–74.9 (nmol/L)	49	1,132	0.85 (0.32–2.26)	0.74	0.76 (0.28–2.02)	0.57	1.00 (0.37–2.68)	0.99
>75.0 (nmol/L)	23	507	1.00 (0.35–2.86)	0.99	0.89 (0.30–2.67)	0.84	1.23 (0.40–3.77)	0.71
**Cadmium**								
**All-cause mortality**								
<0.24 (ug/l)	132	733	1.00 [Reference]		1.00 [Reference]		1.00 [Reference]	
0.24–0.39 (ug/l)	169	769	0.90 (0.64–1.27)	0.55	0.94 (0.66–1.32)	0.70	0.91 (0.66–1.25)	0.56
0.39–0.6 (ug/l)	267	767	1.32 (0.99–1.77)	0.06	1.28 (0.94–1.74)	0.11	1.21 (0.90–1.62)	0.20
>0.6 (ug/l)	240	676	1.96 (1.45–2.64)	<0.001	1.82 (1.33–2.50)	<0.001	1.49 (1.06–2.09)	0.02
**CVD mortality**								
<0.24 (ug/l)	30	733	1.00 [Reference]		1.00 [Reference]		1.00 [Reference]	
0.24–0.39 (ug/l)	44	769	1.21 (0.70–2.11)	0.496	1.22 (0.69–2.17)	0.496	1.16 (0.65–2.07)	0.61
0.39–0.6 (ug/l)	69	767	1.46 (0.75–2.85)	0.26	1.38 (0.71–2.68)	0.33	1.26 (0.65–2.46)	0.49
>0.6 (ug/l)	55	676	2.12 (1.06–4.28)	0.04	1.92 (0.94–3.93)	0.07	1.55 (0.70–3.45)	0.28
**Cancer mortality**								
<0.24 (ug/l)	28	733	1.00 [Reference]		1.00 [Reference]		1.00 [Reference]	
0.24–0.39 (ug/l)	35	769	0.88 (0.40–1.95)	0.75	0.92 (0.42–2.05)	0.85	0.89 (0.43–1.87)	0.76
0.39–0.6 (ug/l)	43	767	0.82 (0.43–1.58)	0.55	0.81 (0.42–1.58)	0.54	0.79 (0.41–1.52)	0.48
>0.6 (ug/l)	48	676	1.95 (0.95–3.98)	0.07	1.85 (0.91–3.77)	0.09	1.21 (0.57–2.59)	0.61
**CRP**								
**All-cause mortality**								
<0.08 (mg/dL)	176	727	1.00 [Reference]		1.00 [Reference]		1.00 [Reference]	
0.08–0.21 (mg/dL)	216	775	1.36 (1.01–1.84)	0.045	1.32 (0.97–1.77)	0.07	1.26 (0.94–1.68)	0.12
0.21–0.49 (mg/dL)	206	708	1.53 (1.21–1.95)	0.001	1.44 (1.12–1.84)	0.01	1.38 (1.08–1.77)	0.01
>0.49 (mg/dL)	210	735	1.90 (1.44–2.52)	<0.001	1.76 (1.34–2.32)	<0.001	1.65 (1.24–2.19)	0.001
**CVD mortality**								
<0.08 (mg/dL)	44	727	1.00 [Reference]		1.00 [Reference]		1.00 [Reference]	
0.08–0.21 (mg/dL)	53	775	1.33 (0.84–2.10)	0.21	1.35 (0.81–2.25)	0.25	1.37 (0.81–2.31)	0.24
0.21–0.49 (mg/dL)	56	708	1.66 (0.84–3.30)	0.14	1.58 (0.76–3.28)	0.22	1.65 (0.76–3.56)	0.20
>0.49 (mg/dL)	45	735	1.37 (0.76–2.47)	0.29	1.26 (0.68–2.34)	0.46	1.24 (0.67–2.31)	0.49
**Cancer mortality**								
<0.08 (mg/dL)	25	727	1.00 [Reference]		1.00 [Reference]		1.00 [Reference]	
0.08–0.21 (mg/dL)	40	775	2.65 (1.46–4.79)	0.002	2.51 (1.40–4.49)	0.002	2.13 (1.18–3.84)	0.01
0.21–0.49 (mg/dL)	44	708	2.98 (1.71–5.17)	<0.001	2.77 (1.58–4.83)	0.001	2.23 (1.27–3.92)	0.01
>0.49 (mg/dL)	45	735	4.25 (2.28–7.90)	<0.001	4.06 (2.23–7.42)	<0.001	3.25 (1.82–5.80)	<0.001

a *Model 1: adjusted for age and sex*.

b *Model 2: Model 1, additionally adjusted for education, marital status, race, the ratio of family income to poverty*.

c *Model 3: Model 2, additionally adjusted for BMI, drinking, smoking, physical activity*.

In the follow-up of people with non-diabetes, 1981 deaths were recorded, including 400 deaths from CVD and 472 deaths from cancer. In the Cox regression analysis, Model 1 adjusts age and gender; Model 2 further adjusts education, marital status, race, the ratio of family income to poverty based on Model 1; Model 3 adjusts BMI, drinking, smoking, physical activity based on Model 2. We used the serum 25(OH)D deficiency group as the reference group, the sufficient serum 25(OH)D group was associated with lower all-cause mortality (HR = 0.67,95%CI = 0.48–0.93, *P* = 0.02) and cardiovascular mortality (HR = 0.50,95%CI = 0.28–0.91, *P* = 0.02). We divided non-diabetic patients into 4 groups based on the interquartile range of cadmium concentration in the blood. At the same time, the low-concentration group (<0.21 μg/l) was used as the control group, the results showed that higher blood cadmium concentration group (>0.6 μg/l) was associated with higher all-cause mortality (HR = 1.70, 95%CI = 1.34–2.15, *P* < 0.001). We divided non-diabetic patients into 4 groups based on the interquartile range of CRP concentration in the blood. At the same time, the low concentration group (<0.08 mg/dL) was used as the control group, the higher CRP concentration group (>0.46 mg/dL) was associated with higher all-cause mortality (HR = 1.62, 95%CI = 1.34–1.96, *P* < 0.001) and cancer mortality (HR = 1.62, 95%CI = 1.15–2.28, *P* = 0.006). Other details were presented in Table 3.

**Table 3:** HR(95%CIs) for all-cause and cause-specific mortality according to serum 25(OH)D,Cadmium and CRP among participants without diabetes.

**Life styles**	**Deaths**	**Total population**	**Model 1^a^**		**Model 2^b^**		**Model 3^c^**	
			**HR (95%CI)**	***P*-value**	**HR (95%CI)**	***P*-value**	**HR (95%CI)**	***P*-value**
**Serum 25 (OH)D**								
**All-cause mortality**								
<25.0 (nmol/L)	88	710	1.00 [Reference]		1.00 [Reference]		1.00 [Reference]	
25.0–49.9 (nmol/L)	624	5,118	0.77 (0.60–0.98)	0.03	0.88 (0.68–1.13)	0.30	0.97 (0.75–1.25)	0.79
50.0–74.9 (nmol/L)	810	6,881	0.51 (0.39–0.68)	<0.001	0.63 (0.47–0.86)	0.004	0.73 (0.54–0.99)	0.04
>75.0 (nmol/L)	459	4,567	0.44 (0.32–0.60)	<0.001	0.57 (0.40–0.80)	0.001	0.67 (0.48–0.93)	0.02
**CVD mortality**								
<25.0 (nmol/L)	18	710	1.00 [Reference]		1.00 [Reference]		1.00 [Reference]	
25.0–49.9 (nmol/L)	128	5,118	0.86 (0.51–1.46)	0.58	0.92 (0.52–1.62)	0.77	0.98 (0.55–1.72)	0.93
50.0–74.9 (nmol/L)	167	6,881	0.51 (0.29–0.87)	0.02	0.56 (0.32–1.00)	0.05	0.62 (0.35–1.11)	0.10
>75.0 (nmol/L)	87	4,567	0.38 (0.22–0.67)	0.001	0.44 (0.24–0.80)	0.01	0.50 (0.28–0.91)	0.02
**Cancer mortality**								
<25.0 (nmol/L)	26	710	1.00 [Reference]		1.00 [Reference]		1.00 [Reference]	
25.0–49.9 (nmol/L)	168	5,118	0.64 (0.40–1.03)	0.07	0.78 (0.49–1.25)	0.31	0.88 (0.54–1.44)	0.62
50.0–74.9 (nmol/L)	170	6,881	0.38 (0.22–0.65)	0.001	0.52 (0.30–0.90)	0.02	0.63 (0.36–1.10)	0.10
>75.0 (nmol/L)	108	4,567	0.33 (0.19–0.56)	<0.001	0.47 (0.27–0.83)	0.01	0.58 (0.33–1.03)	0.06
**Cadmium**								
**All-cause mortality**								
<0.21 (ug/l)	200	4,247	1.00 [Reference]		1.00 [Reference]		1.00 [Reference]	
0.21–0.37 (ug/l)	338	4,506	1.05 (0.82–1.34)	0.71	1.05 (0.83–1.34)	0.68	1.01 (0.80–1.29)	0.92
0.37–0.6 (ug/l)	677	4,273	1.35 (1.06–1.73)	0.02	1.31 (1.03–1.65)	0.03	1.18 (0.94–1.49)	0.15
>0.6 (ug/l)	766	4,250	2.47 (1.99–3.06)	<0.001	2.15 (1.74–2.66)	<0.001	1.70 (1.34–2.15)	<0.001
**CVD mortality**								
<0.21 (ug/l)	42	4,247	1.00 [Reference]		1.00 [Reference]		1.00 [Reference]	
0.21–0.37 (ug/l)	73	4,506	0.89 (0.58–1.38)	0.60	0.90 (0.59–1.39)	0.64	0.88 (0.58–1.35)	0.56
0.37–0.6 (ug/l)	141	4,273	1.17 (0.75–1.81)	0.49	1.14 (0.74–1.77)	0.55	1.04 (0.68–1.62)	0.83
>0.6 (ug/l)	144	4,250	1.67 (1.06–2.63)	0.03	1.49 (0.95–2.34)	0.08	1.26 (0.77–2.08)	0.35
**Cancer mortality**								
<0.21 (ug/l)	47	4,247	1.00 [Reference]		1.00 [Reference]		1.00 [Reference]	
0.21–0.37 (ug/l)	87	4,506	1.46 (0.93–2.31)	0.10	1.45 (0.93–2.27)	0.10	1.35 (0.86–2.12)	0.20
0.37–0.6 (ug/l)	141	4,273	1.66 (1.10–2.51)	0.02	1.61 (1.07–2.41)	0.02	1.34 (0.89–2.02)	0.16
>0.6 (ug/l)	197	4,250	3.37 (2.30–4.96)	<0.001	3.04 (2.06–4.49)	<0.001	2.08 (1.35–3.22)	0.001
**CRP**								
**All-cause mortality**								
<0.08 (mg/dL)	327	4,156	1.00 [Reference]		1.00 [Reference]		1.00 [Reference]	
0.08–0.2 (mg/dL)	525	4,705	1.03 (0.89–1.20)	0.66	1.02 (0.88–1.19)	0.78	1.03 (0.88–1.21)	0.69
0.2–0.46 (mg/dL)	539	4,175	1.22 (1.03–1.45)	0.02	1.19 (0.99–1.43)	0.07	1.18 (0.98–1.43)	0.08
>0.46 (mg/dL)	590	4,240	1.77 (1.49–2.11)	<0.001	1.65 (1.38–1.97)	<0.001	1.62 (1.34–1.96)	<0.001
**CVD mortality**								
<0.08 (mg/dL)	70	4,156	1.00 [Reference]		1.00 [Reference]		1.00 [Reference]	
0.08–0.2 (mg/dL)	112	4,705	1.01 (0.72–1.44)	0.94	1.00 (0.71–1.43)	0.98	0.98 (0.68–1.42)	0.91
0.2–0.46 (mg/dL)	101	4,175	0.99 (0.74–1.31)	0.92	0.97 (0.72–1.29)	0.82	0.92 (0.68–1.24)	0.58
>0.46 (mg/dL)	117	4,240	1.61 (1.12–2.33)	0.01	1.52 (1.05–2.19)	0.03	1.41 (0.97–2.04)	0.07
**Cancer mortality**								
<0.08 (mg/dL)	74	4,156	1.00 [Reference]		1.00 [Reference]		1.00 [Reference]	
0.08–0.2 (mg/dL)	116	4,705	1.05 (0.74–1.48)	0.80	1.03 (0.73–1.44)	0.88	1.00 (0.71–1.40)	0.99
0.2–0.46 (mg/dL)	127	4,175	1.18 (0.87–1.16)	0.28	1.13 (0.83–1.55)	0.44	1.06 (0.76–1.48)	0.73
>0.46 (mg/dL)	155	4,240	1.98 (1.44–2.71)	<0.001	1.79 (1.31–2.47)	<0.001	1.62 (1.15–2.28)	0.01

a *Model 1: adjusted for age and sex*.

b *Model 2: Model 1, additionally adjusted for education, marital status, race, the ratio of family income to poverty*.

c *Model 3: Model 2, additionally adjusted for BMI, drinking, smoking, physical activity*.

### Sensitivity Analyses

In this study, sensitivity analyses were performed on diabetic and non-diabetic patients, excluding the follow-up time of less than 2 years. In diabetic patients, serum 25(OH)D and CRP were associated with all-cause mortality. Cadmium and CRP were associated with all-cause mortality in non-diabetics. Details were in the Supplementary Tables 1, 2.

### Subgroup Analyses

We conducted subgroup analyses of diabetic and non-diabetic people based on age (based on whether the group was older than 65) and gender. serum 25(OH)D and CRP were associated with all-cause mortality in male diabetic patients; serum 25(OH)D, cadmium, and CRP were associated with all-cause mortality in female diabetic patients (Supplementary Table 3). Serum 25(OH)D was associated with all-cause mortality in diabetic patients younger than 65; serum 25(OH)D, cadmium, and CRP were associated with all-cause mortality in diabetic patients older than 65 (Supplementary Table 4). Cadmium and CRP were associated with all-cause mortality in male non-diabetic patients; serum vitamin D, cadmium, and CRP were associated with all-cause mortality in female non-diabetic patients (Supplementary Table 5). Serum 25(OH)D, cadmium, and CRP were associated with all-cause mortality in non-diabetic patients younger than 65; cadmium and CRP were associated with all-cause mortality in non-diabetic patients older than 65 (Supplementary Table 6).

### The Dose-Response Relationship

**Figure 1:**
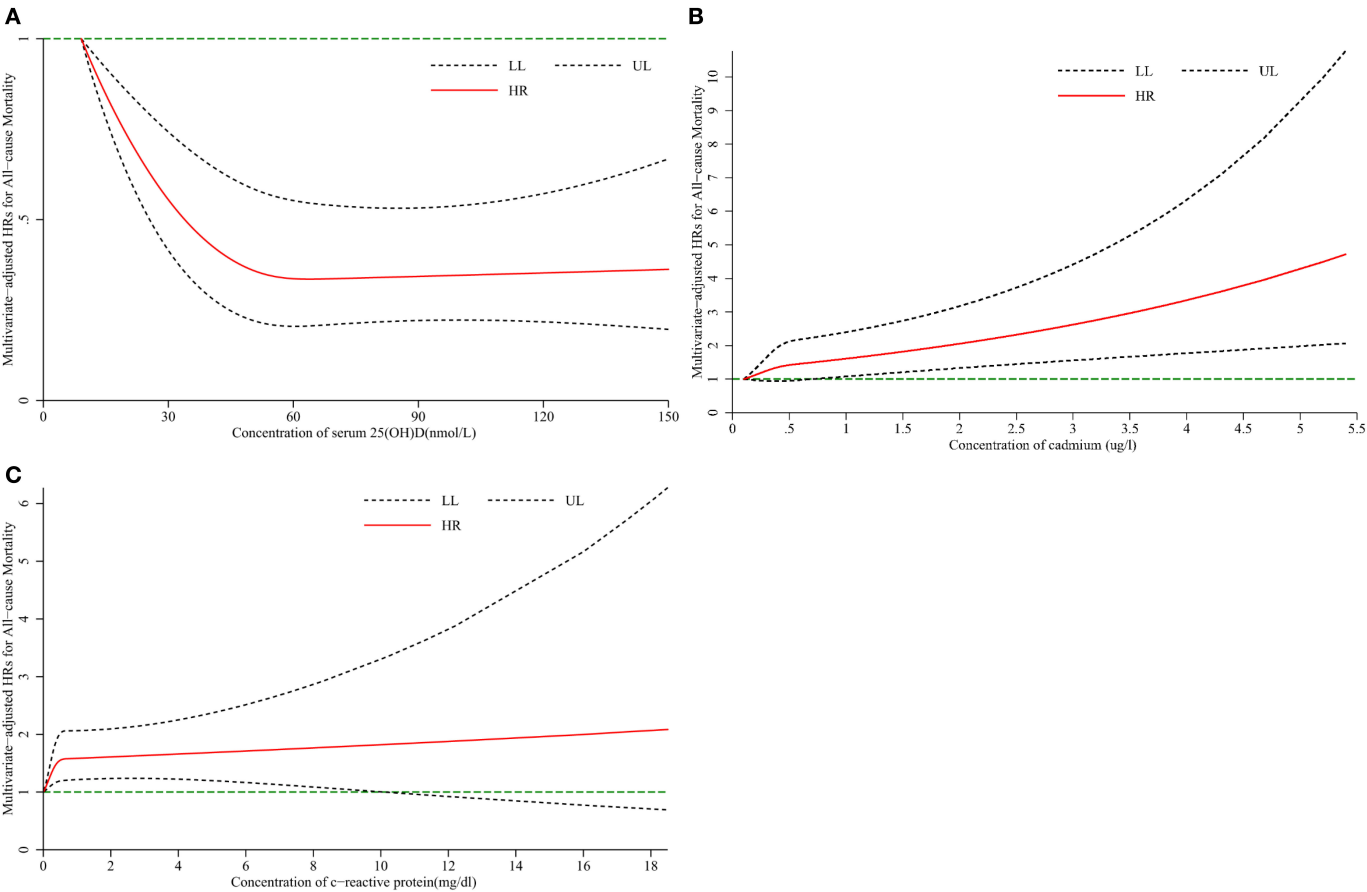
The restricted cubic spline for the association between serum 25(OH)D **(A)**, cadmium **(B)**, C-reactive protein **(C)** and risk of all-cause mortality among adults with diabetes. Knots were placed at the 5th, 50th, and 75th percentiles of the serum 25(OH)D, cadmium, C-reactive protein distribution. Adjustment factors were age, sex, education, marital status, race, the ratio of family income to poverty, BMI, drinking, smoking, physical activity. HR, hazard ratio; LL, lower limit; UL, upper limit.

**Figure 2:**
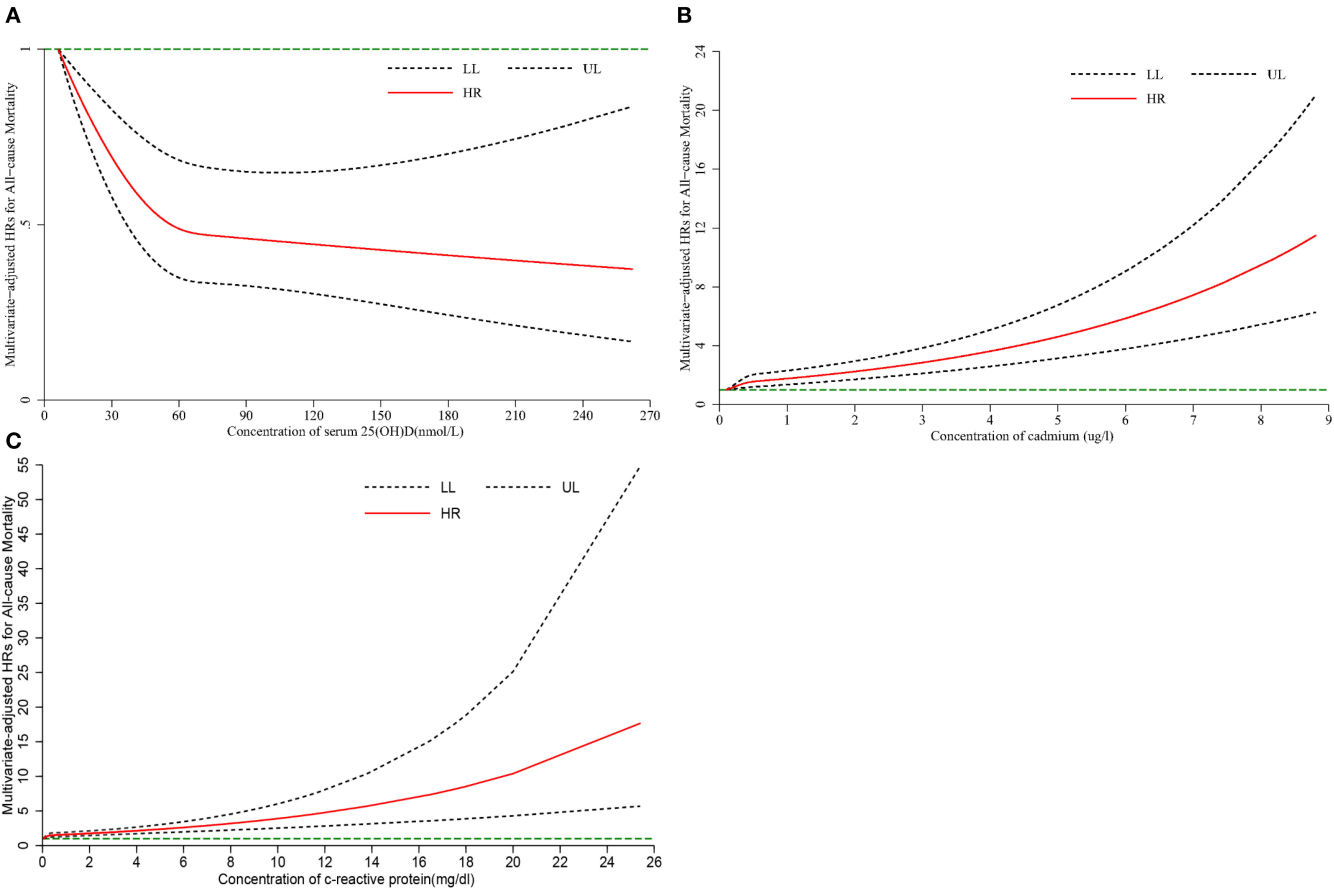
The restricted cubic spline for the association between serum 25(OH)D **(A)**, cadmium **(B)**, C-reactive protein **(C)** and risk of all-cause mortality among adults non-diabetes. Knots were placed at the 5th, 50th, and 75th percentiles of the serum 25(OH)D, cadmium, C-reactive protein distribution. Adjustment factors were age, sex, education, marital status, race, the ratio of family income to poverty, BMI, drinking, smoking, physical activity. HR, hazard ratio; LL, lower limit; UL, upper limit.

After adjusting for multiple covariates, RCS analysis showed that there was a significant nonlinear association between serum 25(OH)D (*P*-nonlinearity <0.001, Figure 1A), cadmium (*P*-nonlinearity = 0.002, Figure 1B), CRP (*P*-nonlinearity = 0.003, Figure 1C), and HR for all-cause mortality risk in diabetic patients. When the serum 25(OH)D concentration is 54.7 nmol/L, HR changes reached a plateau (Figure 1). As the concentration of CRP and cadmium increased, the HR of all-cause mortality increased. There was a significant nonlinear association between serum vitamin D (*P*-nonlinearity <0.001, Figure 2A), cadmium (*P*-nonlinearity <0.001, Figure 2B), CRP (*P*-nonlinearity *P* < 0.001, Figure 2C) and HR for all-cause mortality risk in non-diabetic patients. When the serum 25(OH)D concentration is 60 nmol/L, HR changes reached a plateau (Figure 2). As the concentration of CRP and cadmium increased, the HR of all-cause mortality increased.

## Discussion

In diabetic patients, serum 25(OH)D, cadmium, and CRP were associated with all-cause mortality; serum 25(OH)D was associated with cardiovascular mortality; CRP was associated with cancer mortality. The same conclusions were obtained in non-diabetic patients, but with different levels of risk. Subgroup analyses and sensitivity analyses obtained slightly different results. The results of RCS analyses showed that serum 25(OH)D, cadmium, and CRP had a nonlinear dose-response relationship with the HR of all-cause mortality.

Stratified Mendelian randomization analysis demonstrated a causal relationship between 25(OH)D concentrations and mortality in individuals with low vitamin D status (24). A large cohort study from the UK Biobank, which included 365,530 participants, the results showed that a higher 25(OH)D concentration was non-linearly associated with a lower risk of all-cause, CVD, and cancer mortality. A threshold of 60 nmol/L of 25(OH)D for all-cause and CVD deaths and at 45 nmol/L for cancer deaths, so 45 to 60 nmol/L may represent an intervention goal to reduce the overall risk of premature death (25). In our study, the threshold for diabetic people is 54.7 nmol/L, and the threshold for non-diabetic people is 60 nmol/L. Studies have shown that daily vitamin D supplementation to maintain serum 25(OH)D levels ≥100 nmol/L is a promising approach to reduce the risk of diabetes in adults with prediabetes (26). Studies have shown that in CVD patients, elevated serum 25(OH)D levels are associated with a reduced risk of all-cause and cause-specific mortality (27). Studies have shown that in patients with vitamin D deficiency and no history of myocardial infarction, maintaining (25-OH)D levels at 30 ng/mL at the time of treatment is associated with a lower risk of myocardial infarction (28). A large cohort study in Germany obtained the same results. There was a strong association between 25-hydroxyvitamin D concentration and mortality from all-cause, cardiovascular, cancer, and respiratory diseases (29). The subjects of the two research were the whole population. When we divided the subjects into diabetic and non-diabetic subjects, no correlation was found between serum 25(OH)D and cancer mortality. A study specifically for diabetic patients also confirmed our results. A study from the Third National Health and Nutrition Examination Survey (NHANES III) and NHANES 2001–2014 included 6,329 adult diabetic patients. The results showed that serum 25(OH)D was associated with all-cause mortality and CVD mortality (30). Our conclusions need to be validated by a standardized and large-scale RCT (31).

The Korean National Health and Nutrition Examination Survey (KNHANES), a cross-sectional study based on 10,626 participants aged 20–59 years, found that high blood cadmium levels may be related to stroke and hypertension prevalent in people under 60 (32). A study using data from the National Health and Nutrition Examination Survey (NHANES 1999–2014) showed that there was a potential positive correlation between the concentration of heavy metal mixtures (including cadmium) and overall, CVD, and cancer mortality (33). But the method used is poisson regression analysis and the method we used was cox regression analysis. At the same time, the study emphasizes the effect of multiple metal mixtures on all-cause mortality and specific-cause mortality. Our study paid more attention to the individual effects of cadmium in serum. A study with data from NHANES found that zinc intake may change the association between cadmium and mortality. In addition, the Cd/Zn ratio was positively correlated with all-cause mortality, cancer, and CVD mortality (34). Our research paid more attention to the relationship between serum 25(OH)D, cadmium, and CRP with mortality among people in diabetic and non-diabetic.

A meta-analysis showed that CRP has a non-linear relationship with all-cause mortality and CVD mortality and a linear relationship with cancer and non-cardiovascular mortality (35). Our study also found that CRP had a non-linear relationship with all-cause mortality. A meta-analysis based on a cohort study showed that CRP levels can predict the risk of all-cause mortality and cardiovascular mortality in the general population (36). The indicator calculated by two meta-analyses were RR and our study used HR, which may be the reason for the difference. A study from NHANES (1999–2011) showed that higher levels of CRP were associated with lower overall survival rates and CVD survival rates (37). Our research result was that CRP was associated with all-cause mortality and cancer mortality. The reason may be that we divide the study subjects into diabetic and non-diabetic patients, or the overall level of CRP in this study is low.

This study had the following advantages: Firstly, the study was prospective study design with a larger sample (20,221 participants) and the follow-up time was longer (NAHNEAS 2001–2010, follow-up to 31 December, 2015). Secondly, we analyzed the diabetic and non-diabetic separately. Thirdly, we did both subgroup analyses and sensitivity analyses. Fourthly, we also analyzed the effects of serum 25(OH)D, cadmium, and CRP on all-cause mortality and specific-cause mortality. Several limitations should also be noted. First of all, some statistical indicators were obtained by self-reporting and had bias. Secondly, although we adjusted many confounding factors, other unknown confounding factors may also have an impact on the research results. Finally, this research was designed for observational research, so causality cannot be determined.

## Conclusion

In diabetic patients, serum 25(OH)D, cadmium, and CRP were related to all-cause mortality; serum 25(OH)D was related to cardiovascular mortality; CRP was related to cancer mortality. The similar conclusions were obtained in non-diabetic patients, but with different risk levels. Our conclusions needed to be confirmed and supplemented by follow-up studies.

## Data Availability Statement

The original contributions presented in the study are included in the article/Supplementary Material, further inquiries can be directed to the corresponding author.

## Ethics Statement

The studies involving human participants were reviewed and approved by the NCHS Research Ethics Review Board (ERB). Written informed consent to participate in this study was provided by the participants' legal guardian/next of kin.

## Author Contributions

YL acquired the data, performed the analysis of data, and wrote the manuscript. DY acquired the data and contributed to the analysis of data. FS, FW, XL, SM, and HW contributed to the coding of the statistical analysis. CY designed and evaluated the whole work. All authors read and approved the final manuscript.

## Funding

This research was funded by the National Natural Science Foundation of China (Grant Nos. 81773552 and 82173626).

## Conflict of Interest

The authors declare that the research was conducted in the absence of any commercial or financial relationships that could be construed as a potential conflict of interest.

## Publisher's Note

All claims expressed in this article are solely those of the authors and do not necessarily represent those of their affiliated organizations, or those of the publisher, the editors and the reviewers. Any product that may be evaluated in this article, or claim that may be made by its manufacturer, is not guaranteed or endorsed by the publisher.

## References

[1] Saeedi P Petersohn I Salpea P Malanda B Karuranga S Unwin N. Global and regional diabetes prevalence estimates for 2019 and projections for 2030 and 2045: results from the International Diabetes Federation Diabetes Atlas, 9(th) edition. Diabetes Res Clin Pract. (2019) 157:107843. 10.1016/j.diabres.2019.107843 31518657

[2] Dal Canto E Ceriello A Rydén L Ferrini M Hansen TB Schnell O. Diabetes as a cardiovascular risk factor: an overview of global trends of macro and micro vascular complications. Eur J Prev Cardiol. (2019) 26:25–32. 10.1177/2047487319878371 31722562

[3] Wang L Manson JE Song Y Sesso HD. Systematic review: vitamin D and calcium supplementation in prevention of cardiovascular events. Ann Intern Med. (2010) 152:315–23. 10.7326/0003-4819-152-5-201003020-00010 20194238

[4] Dimitrakopoulou VI Tsilidis KK Haycock PC Dimou NL Al-Dabhani K Martin RM. Circulating vitamin D concentration and risk of seven cancers: mendelian randomisation study. BMJ. (2017) 359:j4761. 10.1136/bmj.j4761 29089348 PMC5666592

[5] Keum N Lee DH Greenwood DC Manson JE Giovannucci E. Vitamin D supplementation and total cancer incidence and mortality: a meta-analysis of randomized controlled trials. Ann Oncol. (2019) 30:733–43. 10.1093/annonc/mdz059 30796437 PMC6821324

[6] Zhang Y Fang F Tang J Jia L Feng Y Xu P. Association between vitamin D supplementation and mortality: systematic review and meta-analysis. BMJ. (2019) 366:l4673. 10.1136/bmj.l4673 31405892 PMC6689821

[7] Bjelakovic G Gluud LL Nikolova D Whitfield K Wetterslev J Simonetti RG. Vitamin D supplementation for prevention of mortality in adults. Cochrane Database Syst Rev. (2021) 2014:CD007470. 10.1002/14651858.CD007470.pub3 24414552 PMC11285307

[8] Joergensen C Gall MA Schmedes A Tarnow L Parving HH Rossing P. Vitamin D levels and mortality in type 2 diabetes. Diabetes Care. (2010) 33:2238–2243. 10.2337/dc10-0582 20606205 PMC2945166

[9] Jennersjö P Guldbrand H Björne S Länne T Fredrikson M Lindström T. A prospective observational study of all-cause mortality in relation to serum 25-OH vitamin D3 and parathyroid hormone levels in patients with type 2 diabetes. Diabetol Metab Syndr. (2015) 7:53. 10.1186/s13098-015-0049-9 26078787 PMC4466811

[10] Kim K Melough MM Vance TM Noh H Koo SI Chun OK. Dietary cadmium intake and sources in the US. Nutrients. (2018) 11:2. 10.3390/nu11010002 30577418 PMC6356330

[11] Schaefer HR Dennis S Fitzpatrick S. Cadmium: mitigation strategies to reduce dietary exposure. J Food Sci. (2020) 85:260–267. 10.1111/1750-3841.14997 31957884 PMC7027482

[12] Nordberg G Jin T Wu X Lu J Chen L Liang Y. Kidney dysfunction and cadmium exposure–factors influencing dose-response relationships. J Trace Elements Med. (2012) 26:197–200. 10.1016/j.jtemb.2012.03.007 22565016

[13] James KA Meliker JR. Environmental cadmium exposure and osteoporosis: a review. Int J Public Health. (2013) 58:737–45. 10.1007/s00038-013-0488-8 23877535

[14] Messner B Knoflach M Seubert A Ritsch A Pfaller K Henderson B. Cadmium is a novel and independent risk factor for early atherosclerosis mechanisms and in vivo relevance. Arterioscler Thromb Vasc Biol. (2009) 29:1392–8. 10.1161/ATVBAHA.109.190082 19556524

[15] Lee MS Park SK Hu H Lee S. Cadmium exposure and cardiovascular disease in the 2005 Korea National Health and Nutrition Examination Survey. Environ Res. (2011) 111:171–6. 10.1016/j.envres.2010.10.006 21055738 PMC3683977

[16] Larsson SC Wolk A. Urinary cadmium and mortality from all causes, cancer and cardiovascular disease in the general population: systematic review and meta-analysis of cohort studies. Int J Epidemiol. (2016) 45:782–791. 10.1093/ije/dyv086 25997435

[17] Rajab IM Hart PC Potempa LA. How C-reactive protein structural isoforms with distinctive bioactivities affect disease progression. Front Immunol. (2020) 11:2126. 10.3389/fimmu.2020.02126 33013897 PMC7511658

[18] Markozannes G Koutsioumpa C Cividini S Monori G Tsilidis KK Kretsavos N. Global assessment of C-reactive protein and health-related outcomes: an umbrella review of evidence from observational studies and Mendelian randomization studies. Eur J Epidemiol. (2021) 36:11–36. 10.1007/s10654-020-00681-w 32978716 PMC7847446

[19] Shen Y Zhang Y Xiong S Zhu X Ke C. High-sensitivity C-reactive protein and cystatin C independently and jointly predict all-cause mortality among the middle-aged and elderly Chinese population. Clin Biochem. (2019) 65:7–14. 10.1016/j.clinbiochem.2018.12.012 30592989

[20] Maluf CB Barreto SM Giatti L Ribeiro AL Vidigal PG Azevedo DRM. Association between C reactive protein and all-cause mortality in the ELSA-Brasil cohort. J Epidemiol Commun Health. (2020) 74:421–7. 10.1136/jech-2019-213289 32102838 PMC7307658

[21] Tian R Tian M Wang L Qian H Zhang S Pang H. C-reactive protein for predicting cardiovascular and all-cause mortality in type 2 diabetic patients: A meta-analysis. Cytokine. (2019) 117:59–64. 10.1016/j.cyto.2019.02.005 30826600

[22] Bull FC Al-Ansari SS Biddle S Borodulin K Buman MP Cardon G. World Health Organization 2020 guidelines on physical activity and sedentary behaviour. Br J Sports Med. (2020) 54:1451–62. 10.1136/bjsports-2020-102955 33239350 PMC7719906

[23] Holick MF Binkley NC Bischoff-Ferrari HA Gordon CM Hanley DA Heaney RP. Evaluation, treatment, and prevention of vitamin D deficiency: an Endocrine Society clinical practice guideline. J Clin Endocrinol Metab. (2011) 96:1911–30. 10.1210/jc.2011-0385 21646368

[24] Emerging Risk Factors Collaboration/EPIC-CVD/Vitamin D Studies Collaboration. Estimating dose-response relationships for vitamin D with coronary heart disease, stroke, and all-cause mortality: observational and Mendelian randomisation analyses. Lancet Diabetes Endocrinol. (2021) 9:837–46. 10.1016/S2213-8587(21)00263-1 34717822 PMC8600124

[25] Fan X Wang J Song M Giovannucci EL Ma H Jin G. Vitamin D status and risk of all-cause and cause-specific mortality in a large cohort: results from the UK biobank. J Clin Endocrinol Metab. (2020) 105:432. 10.1210/clinem/dgaa432 32620963

[26] Dawson-Hughes B Staten MA Knowler WC Nelson J Vickery EM LeBlanc ES. Intratrial exposure to vitamin D and new-onset diabetes among adults with prediabetes: a secondary analysis from the vitamin D and type 2 diabetes (D2D) study. Diabetes Care. (2020) 43:2916–22. 10.2337/dc20-1765 33020052 PMC7770274

[27] Dai L Liu M Chen L. Association of serum 25-hydroxyvitamin D concentrations with all-cause and cause-specific mortality among adult patients with existing cardiovascular disease. Front Nutr. (2021) 8:740855. 10.3389/fnut.2021.740855 34631770 PMC8496747

[28] Acharya P Dalia T Ranka S Sethi P Oni OA Safarova MS. The effects of vitamin D supplementation and 25-Hydroxyvitamin D levels on the risk of myocardial infarction and mortality. J Endocr Soc. (2021) 5:bvab124. 10.1210/jendso/bvab124 34396023 PMC8358990

[29] Schöttker B Haug U Schomburg L Köhrle J Perna L Müller H. Strong associations of 25-hydroxyvitamin D concentrations with all-cause, cardiovascular, cancer, and respiratory disease mortality in a large cohort study. Am J Clin Nutr. (2013) 97:782–93. 10.3945/ajcn.112.047712 23446902

[30] Wan Z Guo J Pan A Chen C Liu L Liu G. Association of serum 25-hydroxyvitamin D concentrations with all-cause and cause-specific mortality among individuals with diabetes. Diabetes Care. (2021) 44:350–7. 10.2337/dc20-1485 33168652

[31] Pilz S Trummer C Theiler-Schwetz V Grübler MR Verheyen ND Odler B. Critical appraisal of large vitamin D randomized controlled trials. Nutrients. (2022) 14:303. 10.3390/nu14020303 35057483 PMC8778517

[32] Jeong J Yun SM Kim M Koh YH. Association of blood cadmium with cardiovascular disease in Korea: from the Korea National Health and Nutrition Examination Survey 2008-2013 and 2016. Int J Environ Res Public Health. (2020) 17:288. 10.3390/ijerph17176288 32872339 PMC7503499

[33] Duan W Xu C Liu Q Xu J Weng Z Zhang X. Levels of a mixture of heavy metals in blood and urine and all-cause, cardiovascular disease and cancer mortality: a population-based cohort study. Environ Pollution. (2020) 263:114630. 10.1016/j.envpol.2020.114630 33618481

[34] Kim K Melough MM Sakaki JR Ha K Marmash D Noh H. Association between urinary cadmium to zinc intake ratio with adult mortality in a follow-Up Study of NHANES 1988-1994 and 1999-2004. Nutrients. (2019) 12:56. 10.3390/nu12010056 31878194 PMC7019386

[35] Ni P Yu M Zhang R Cheng C He M Wang H. Dose-response association between C-reactive protein and risk of all-cause and cause-specific mortality: a systematic review and meta-analysis of cohort studies. Ann Epidemiol. (2020) 51:20–27.e11. 10.1016/j.annepidem.2020.07.005 32702432

[36] Li Y Zhong X Cheng G Zhao C Zhang L Hong Y. Hs-CRP and all-cause, cardiovascular, and cancer mortality risk: a meta-analysis. Atherosclerosis. (2017) 259:75–82. 10.1016/j.atherosclerosis.2017.02.003 28327451

[37] Liu J Zhang Y Lavie CJ Tabung FK Xu J Hu Q. Associations of C-reactive protein and fibrinogen with mortality from all-causes, cardiovascular disease and cancer among U.S. adults. Prevent Med. (2020) 139:106044. 10.1016/j.ypmed.2020.106044 32097752

