# Supplementary material for: Association of Serum 25(OH)D, Cadmium, CRP With All-Cause, Cause-Specific Mortality: A Prospective Cohort Study
Source: Front Nutr. 2022 Apr 27;9:803985. doi: 10.3389/fnut.2022.803985 (PMC9094577; doi:10.3389/fnut.2022.803985)
Supplement: Supplementary file 1 [file Data_Sheet_1.docx]

**Table S1 Sensitivity analysis on the association between serum 25(OH)D，Cadmium , CRP and all-cause mortality among participants with diabetes**

| **All-cause mortality** | **Deaths** | **Total population** | **Model 1 ^a^ Model 2 ^b^  Model 3^c^** | | | | | |
| --- | --- | --- | --- | --- | --- | --- | --- | --- |
|  |  |  | **HR (95%CI)** | **P value** | **HR (95%CI)** | **P value** | **HR (95%CI)** | **P value** |
| **Serum 25(OH)D** |  |  |  |  |  |  |  |  |
| <25.0(nmol/L) | 55 | 178 | 1.00 [Reference] |  | 1.00 [Reference] |  | 1.00[Reference] |  |
| 25.0–49.9(nmol/L) | 248 | 1064 | 0.50(0.34- 0.73) | 0.001 | 0.46(0.30-0.69) | <0.001 | 0.48(0.32-0.70) | <0.001 |
| 50.0–74.9(nmol/L) | 261 | 1090 | 0.37(0.25-0.54) | <0.001 | 0.33(0.22-0.50) | <0.001 | 0.37(0.24-0.56) | <0.001 |
| >75.0(nmol/L) | 115 | 484 | 0.37(0.26-0.54) | <0.001 | 0.34(0.23-0.52) | <0.001 | 0.37(0.24-0.57) | <0.001 |
| **Cadmium** |  |  |  |  |  |  |  |  |
| <0.23(ug/l) | 109 | 680 | 1.00 [Reference] |  | 1.00 [Reference] |  | 1.00[Reference] |  |
| 0.23-0.38(ug/l) | 140 | 742 | 0.90(0.63-1.28) | 0.54 | 0.94(0.65-1.35) | 0.73 | 0.90(0.63-1.29) | 0.56 |
| 0.38-0.6(ug/l) | 233 | 761 | 1.31(0.93-1.83) | 0.12 | 1.29(0.90-1.85) | 0.17 | 1.18(0.83-1.68) | 0.35 |
| >0.6(ug/l) | 197 | 633 | 1.94(1.41-2.68) | <0.001 | 1.83(1.31-2.55) | 0.001 | 1.43(0.97-2.11) | 0.070 |
| **CRP** |  |  |  |  |  |  |  |  |
| <0.13(mg/dL) | 145 | 661 | 1.00 [Reference] |  | 1.00 [Reference] |  | 1.00 [Reference] |  |
| 0.13-0.32(mg/dL) | 191 | 758 | 1.43(1.04-1.96) | 0.03 | 1.37(1.02-1.84) | 0.04 | 1.32(0.98-1.76) | 0.07 |
| 0.32-0.69(mg/dL) | 176 | 697 | 1.48(1.15-1.90) | 0.003 | 1.40(1.09-1.79) | 0.01 | 1.34(1.03-1.73) | 0.03 |
| >0.69(mg/dL) | 167 | 700 | 1.79(1.32-2.43) | <0.001 | 1.68(1.26-2.24) | 0.001 | 1.56(1.17-2.09) | 0.003 |

^a^ Model 1: adjusted for age and sex

^b^ Model 2: Model 1, additionally adjusted for education, marital status, race, ratio of family income to poverty

^c^ Model 3: Model 2, additionally adjusted for BMI, drinking, smoking, stroke, physical activity

Sensitivity analysis: excluding subjects who have been followed up for less than 2 years.

**Table S2 Sensitivity analysis on the association between serum 25(OH)D，Cadmium , CRP and all-cause mortality among participants with non-diabetes**

| **All-cause mortality** | **Deaths** | **Total population** | **Model 1 ^a^ Model 2 ^b^  Model 3^c^** | | | | | |
| --- | --- | --- | --- | --- | --- | --- | --- | --- |
|  |  |  | **HR (95%CI)** | **P value** | **HR (95%CI)** | **P value** | **HR (95%CI)** | **P value** |
| **Serum 25(OH)D** |  |  |  |  |  |  |  |  |
| <25.0(nmol/L) | 66 | 688 | 1.00 [Reference] |  | 1.00 [Reference] |  | 1.00[Reference] |  |
| 25.0–49.9(nmol/L) | 519 | 5013 | 0.84(0.63-1.13) | 0.25 | 0.97(0.72-1.30) | 0.82 | 1.07(0.79-1.46) | 0.65 |
| 50.0–74.9(nmol/L) | 715 | 6786 | 0.59(0.43-0.81) | 0.001 | 0.73(0.52-1.02) | 0.06 | 0.84(0.60-1.18) | 0.32 |
| >75.0(nmol/L) | 393 | 4501 | 0.50(0.36-0.69) | <0.001 | 0.64(0.44-0.92) | 0.02 | 0.75(0.52-1.08) | 0.12 |
| **Cadmium** |  |  |  |  |  |  |  |  |
| <0.21(ug/l) | 175 | 4222 | 1.00 [Reference] |  | 1.00 [Reference] |  | 1.00[Reference] |  |
| 0.21-0.36(ug/l) | 270 | 4322 | 1.02(0.78-1.33) | 0.90 | 1.03(0.79-1.33) | 0.84 | 0.98(0.76-1.28) | 0.91 |
| 0.36-0.6(ug/l) | 609 | 4321 | 1.37(1.05-1.78) | 0.02 | 1.32(1.02-1.71) | 0.03 | 1.20(0.93-1.54) | 0.16 |
| >0.6(ug/l) | 639 | 4123 | 2.46(1.97-3.08) | <0.001 | 2.16(1.73-2.69) | <0.001 | 1.69(1.32-2.17) | <0.001 |
| **CRP** |  |  |  |  |  |  |  |  |
| <0.08(mg/dL) | 298 | 4127 | 1.00 [Reference] |  | 1.00 [Reference] |  | 1.00 [Reference] |  |
| 0.08-0.2(mg/dL) | 453 | 4633 | 0.98(0.84-1.15) | 0.80 | 0.98(0.83-1.15) | 0.76 | 0.98(0.83-1.16) | 0.80 |
| 0.2-0.45(mg/dL) | 471 | 4019 | 1.20(1.00-1.45) | 0.048 | 1.17(0.96-1.43) | 0.11 | 1.16(0.95-1.42) | 0.15 |
| >0.45(mg/dL) | 471 | 4209 | 1.53(1.25-1.86) | <0.001 | 1.44(1.18-1.75) | <0.001 | 1.40(1.13-1.74) | 0.003 |

^a^ Model 1: adjusted for age and sex

^b^ Model 2: Model 1, additionally adjusted for education, marital status, race, ratio of family income to poverty

^c^ Model 3: Model 2, additionally adjusted for BMI, drinking, smoking, stroke, physical activity

Sensitivity analysis: excluding subjects who have been followed up for less than 2 years.

**Table S3 Subgroup analysis for gender on the association between serum 25(OH)D，Cadmium , CRP and all-cause mortality among participants with diabetes**

| **All-cause mortality（Male）** | **Deaths** | **Total population** | **Model 1 ^a^ Model 2 ^b^  Model 3^c^** | | | | | |
| --- | --- | --- | --- | --- | --- | --- | --- | --- |
|  |  |  | **HR (95%CI)** | **P value** | **HR (95%CI)** | **P value** | **HR (95%CI)** | **P value** |
| **Serum 25(OH)D** |  |  |  |  |  |  |  |  |
| <25.0(nmol/L) | 23 | 74 | 1.00 [Reference] |  | 1.00 [Reference] |  | 1.00[Reference] |  |
| 25.0–49.9(nmol/L) | 165 | 574 | 0.63(0.32-1.25) | 0.18 | 0.59(0.29-1.17) | 0.13 | 0.58(0.28-1.19) | 0.14 |
| 50.0–74.9(nmol/L) | 200 | 660 | 0.47(0.25-0.87) | 0.02 | 0.40(0.21-0.77) | 0.01 | 0.43(0.21-0.88) | 0.02 |
| >75.0(nmol/L) | 85 | 257 | 0.54(0.28-1.04) | 0.06 | 0.47(0.23-0.93) | 0.03 | 0.50(0.24-1.04) | 0.07 |
| **Cadmium** |  |  |  |  |  |  |  |  |
| <0.21 (ug/l) | 89 | 391 | 1.00 [Reference] |  | 1.00 [Reference] |  | 1.00[Reference] |  |
| 0.21-0.34(ug/l) | 87 | 393 | 0.88(0.58-1.34) | 0.54 | 0.93(0.61-1.42) | 0.73 | 0.87(0.58-1.29) | 0.48 |
| 0.34-0.6(ug/l) | 159 | 425 | 1.31(0.91-1.88) | 0.15 | 1.31(0.89-1.92) | 0.17 | 1.24(0.86-1.79) | 0.25 |
| >0.6(ug/l) | 138 | 356 | 1.87(1.30-2.70) | 0.001 | 1.76(1.18-2.61) | 0.006 | 1.43(0.93-2.20) | 0.10 |
| **CRP** |  |  |  |  |  |  |  |  |
| <0.11(mg/dL) | 85 | 361 | 1.00 [Reference] |  | 1.00 [Reference] |  | 1.00 [Reference] |  |
| 0.11-0.26(mg/dL) | 126 | 428 | 1.44(0.94-2.22) | 0.10 | 1.38(0.91-2.09) | 0.13 | 1.31(0.85-2.01) | 0.21 |
| 0.26-0.57(mg/dL) | 130 | 404 | 1.54(1.10-2.14) | 0.01 | 1.51(1.06-2.14) | 0.02 | 1.45(1.03-2.04) | 0.03 |
| >0.57(mg/dL) | 132 | 372 | 1.99(1.34-2.95) | 0.001 | 1.83(1.25-2.67) | 0.002 | 1.70(1.15-2.51) | 0.01 |
| **All-cause mortality（Female）** |  |  |  |  |  |  |  |  |
| **Serum 25(OH)D** |  |  |  |  |  |  |  |  |
| <25.0(nmol/L) | 42 | 114 | 1.00 [Reference] |  | 1.00 [Reference] |  | 1.00[Reference] |  |
| 25.0–49.9(nmol/L) | 137 | 544 | 0.54(0.34-0.86) | 0.01 | 0.52(0.32-0.83) | 0.01 | 0.56(0.36-0.87) | 0.01 |
| 50.0–74.9(nmol/L) | 103 | 472 | 0.35(0.23-0.53) | <0.001 | 0.34(0.22-0.54) | <0.001 | 0.39(0.24-0.62) | <0.001 |
| >75.0(nmol/L) | 53 | 250 | 0.29(0.18-0.45) | <0.001 | 0.30(0.18-0.50) | <0.001 | 0.32(0.19-0.55) | <0.001 |
| **Cadmium** |  |  |  |  |  |  |  |  |
| <0.27(ug/l) | 43 | 328 | 1.00 [Reference] |  | 1.00 [Reference] |  | 1.00[Reference] |  |
| 0.27-0.4(ug/l) | 100 | 430 | 1.08(0.71-1.64) | 0.72 | 1.10(0.72-1.68) | 0.65 | 1.12(0.74-1.70) | 0.58 |
| 0.4-0.6(ug/l) | 90 | 302 | 1.60(1.10-2.33) | 0.01 | 1.63(1.12-2.38) | 0.01 | 1.45(0.98-2.14) | 0.06 |
| >0.6(ug/l) | 102 | 320 | 2.33(1.51-3.57) | <0.001 | 2.23(1.45-3.42) | <0.001 | 1.80(1.06-3.05) | 0.029 |
| **CRP** |  |  |  |  |  |  |  |  |
| <0.17(mg/dL) | 86 | 327 | 1.00 [Reference] |  | 1.00 [Reference] |  | 1.00 [Reference] |  |
| 0.17-0.42(mg/dL) | 80 | 366 | 1.11(0.79-1.56) | 0.55 | 1.01(0.70-1.44) | 0.97 | 0.98(0.67-1.42) | 0.90 |
| 0.42-0.88(mg/dL) | 84 | 343 | 1.38(0.97-1.96) | 0.07 | 1.31(0.92-1.86) | 0.13 | 1.39(0.97-1.99) | 0.07 |
| >0.88(mg/dL) | 85 | 344 | 1.97(1.36-2.87) | 0.001 | 1.80(1.22-2.66) | 0.004 | 1.66(1.09-2.53) | 0.02 |

^a^ Model 1: adjusted for age

^b^ Model 2: Model 1, additionally adjusted for education, marital status, race, ratio of family income to poverty

^c^ Model 3: Model 2, additionally adjusted for BMI, drinking, smoking, stroke, physical activity

**Table S4 Subgroup analysis for age on the association between serum 25(OH)D，Cadmium , CRP and all-cause mortality among participants with diabetes**

| **All-cause mortality（<65）** | **Deaths** | **Total population** | **Model 1 ^a^ Model 2 ^b^  Model 3^c^** | | | | | |
| --- | --- | --- | --- | --- | --- | --- | --- | --- |
|  |  |  | **HR (95%CI)** | **P value** | **HR (95%CI)** | **P value** | **HR (95%CI)** | **P value** |
| **Serum 25(OH)D** |  |  |  |  |  |  |  |  |
| <25.0(nmol/L) | 29 | 123 | 1.00 [Reference] |  | 1.00 [Reference] |  | 1.00[Reference] |  |
| 25.0–49.9(nmol/L) | 99 | 682 | 0.53(0.29-0.98) | 0.045 | 0.48(0.26-0.87) | 0.02 | 0.55(0.31-0.99) | 0.045 |
| 50.0–74.9(nmol/L) | 66 | 581 | 0.30(0.17-0.53) | <0.001 | 0.25(0.14-0.47) | <0.001 | 0.31(0.15-0.61) | 0.001 |
| >75.0(nmol/L) | 20 | 204 | 0.38(0.19-0.78) | 0.01 | 0.34(0.16-0.70) | 0.004 | 0.36(0.17-0.76) | 0.01 |
| **Cadmium** |  |  |  |  |  |  |  |  |
| <0.2 (ug/l) | 22 | 291 | 1.00 [Reference] |  | 1.00 [Reference] |  | 1.00[Reference] |  |
| 0.2-0.34(ug/l) | 44 | 520 | 0.67(0.32-1.42) | 0.29 | 0.70(0.33-1.48) | 0.35 | 0.62(0.31-1.26) | 0.18 |
| 0.34-0.6(ug/l) | 63 | 398 | 1.58(0.79-3.15) | 0.19 | 1.60(0.81-1.36) | 0.18 | 1.38(0.76-2.50) | 0.29 |
| >0.6(ug/l) | 85 | 381 | 2.65(1.44-4.87) | 0.002 | 2.29(1.24-4.22) | 0.01 | 1.61(0.80-3.24) | 0.18 |
| **CRP** |  |  |  |  |  |  |  |  |
| <0.16(mg/dL) | 44 | 388 | 1.00 [Reference] |  | 1.00 [Reference] |  | 1.00 [Reference] |  |
| 0.16-0.38(mg/dL) | 59 | 416 | 1.44(0.87-2.40) | 0.16 | 1.41(0.83-2.39) | 0.20 | 1.45(0.90-2.32) | 0.12 |
| 0.38-0.82(mg/dL) | 57 | 395 | 1.29(0.79-2.11) | 0.31 | 1.13(0.69-1.84) | 0.63 | 1.13(0.73-1.74) | 0.57 |
| >0.82(mg/dL) | 54 | 391 | 1.77(1.02-3.09) | 0.04 | 1.63(0.92-2.87) | 0.09 | 1.56(0.89-2.75) | 0.12 |
| **All-cause mortality（≥65）** |  |  |  |  |  |  |  |  |
| **Serum 25(OH)D** |  |  |  |  |  |  |  |  |
| <25.0(nmol/L) | 36 | 65 | 1.00 [Reference] |  | 1.00 [Reference] |  | 1.00[Reference] |  |
| 25.0–49.9(nmol/L) | 203 | 436 | 0.62(0.42-0.91) | 0.02 | 0.60(0.38-0.93) | 0.02 | 0.61(0.39-0.93) | 0.02 |
| 50.0–74.9(nmol/L) | 237 | 551 | 0.48(0.32-0.71) | <0.001 | 0.45(0.28-0.71) | 0.001 | 0.46(0.29-0.74) | 0.001 |
| >75.0(nmol/L) | 118 | 303 | 0.43(0.29-0.64) | <0.001 | 0.42(0.26-0.67) | <0.001 | 0.44(0.26-0.73) | 0.002 |
| **Cadmium** |  |  |  |  |  |  |  |  |
| <0.29(ug/l) | 119 | 332 | 1.00 [Reference] |  | 1.00 [Reference] |  | 1.00[Reference] |  |
| 0.29-0.4(ug/l) | 164 | 404 | 1.02(0.77-1.35) | 0.88 | 1.02(0.77-1.35) | 0.88 | 1.04(0.80-1.35) | 0.78 |
| 0.4-0.6(ug/l) | 156 | 324 | 1.22(0.96-1.56) | 0.10 | 1.21(0.93-1.58) | 0.15 | 1.15(0.89-1.50) | 0.28 |
| >0.6(ug/l) | 155 | 295 | 1.58(1.19-2.10) | 0.002 | 1.53(1.14-2.04) | 0.01 | 1.43(1.01-2.03) | 0.04 |
| **CRP** |  |  |  |  |  |  |  |  |
| <0.11(mg/dL) | 108 | 308 | 1.00 [Reference] |  | 1.00 [Reference] |  | 1.00 [Reference] |  |
| 0.11-0.27(mg/dL) | 153 | 380 | 1.33(0.97-1.83) | 0.08 | 1.29(0.94-1.77) | 0.11 | 1.26(0.92-1.71) | 0.15 |
| 0.27-0.56(mg/dL) | 156 | 332 | 1.64(1.21-2.22) | 0.002 | 1.57(1.15-2.15) | 0.01 | 1.49(1.10-2.03) | 0.01 |
| >0.56(mg/dL) | 177 | 335 | 2.05(1.52-2.77) | <0.001 | 1.93(1.43-2.61) | <0.001 | 1.82(1.32-2.50) | <0.001 |

^a^ Model 1: adjusted for gender

^b^ Model 2: Model 1, additionally adjusted for education, marital status, race, ratio of family income to poverty

^c^ Model 3: Model 2, additionally adjusted for BMI, drinking, smoking, stroke, physical activity

**Table S5 Subgroup analysis for gender on the association between serum 25(OH)D，Cadmium , CRP and all-cause mortality among participants with non-diabetes**

| **All-cause mortality（Male）** | **Deaths** | **Total population** | **Model 1 ^a^ Model 2 ^b^  Model 3^c^** | | | | | |
| --- | --- | --- | --- | --- | --- | --- | --- | --- |
|  |  |  | **HR (95%CI)** | **P value** | **HR (95%CI)** | **P value** | **HR (95%CI)** | **P value** |
| **Serum 25(OH)D** |  |  |  |  |  |  |  |  |
| <25.0(nmol/L) | 39 | 272 | 1.00 [Reference] |  | 1.00 [Reference] |  | 1.00[Reference] |  |
| 25.0–49.9(nmol/L) | 358 | 2369 | 0.88(0.62-1.26) | 0.49 | 1.09(0.77-1.54) | 0.64 | 1.18(0.82-1.70) | 0.36 |
| 50.0–74.9(nmol/L) | 484 | 3647 | 0.55(0.38-0.79) | 0.002 | 0.76(0.53-1.08) | 0.13 | 0.83(0.57-1.22) | 0.35 |
| >75.0(nmol/L) | 264 | 2042 | 0.50(0.34-0.73) | <0.001 | 0.71(0.48-1.06) | 0.09 | 0.80(0.54-1.20) | 0.28 |
| **Cadmium** |  |  |  |  |  |  |  |  |
| <0.2 (ug/l) | 56 | 1487 | 1.00 [Reference] |  | 1.00 [Reference] |  | 1.00[Reference] |  |
| 0.2-0.33(ug/l) | 250 | 2743 | 1.26(0.87-1.84) | 0.22 | 1.22(0.83-1.79) | 0.30 | 1.20(0.82-1.74) | 0.35 |
| 0.33-0.6(ug/l) | 406 | 2018 | 1.85(1.27-2.68) | 0.002 | 1.67(1.14-2.44) | 0.01 | 1.51(1.04-2.21) | 0.03 |
| >0.6(ug/l) | 433 | 2082 | 3.46(2.30-5.21) | <0.001 | 2.73(1.78-4.17) | <0.001 | 2.22(1.42-3.49) | 0.001 |
| **CRP** |  |  |  |  |  |  |  |  |
| <0.07(mg/dL) | 168 | 1984 | 1.00 [Reference] |  | 1.00 [Reference] |  | 1.00 [Reference] |  |
| 0.07-0.16(mg/dL) | 251 | 2314 | 1.00(0.77-1.30) | 0.98 | 1.03(0.79-1.35) | 0.83 | 1.05(0.81-1.36) | 0.70 |
| 0.16-0.34(mg/dL) | 302 | 1959 | 1.23(0.98-1.55) | 0.07 | 1.21(0.95-1.54) | 0.12 | 1.14(0.90-1.44) | 0.28 |
| >0.34(mg/dL) | 424 | 2073 | 1.92(1.51-2.44) | <0.001 | 1.80(1.40-2.31) | <0.001 | 1.68(1.31-2.15) | <0.001 |
| **All-cause mortality（Female）** |  |  |  |  |  |  |  |  |
| **Serum 25(OH)D** |  |  |  |  |  |  |  |  |
| <25.0(nmol/L) | 49 | 438 | 1.00 [Reference] |  | 1.00 [Reference] |  | 1.00[Reference] |  |
| 25.0–49.9(nmol/L) | 266 | 2749 | 0.68(0.48-0.96) | 0.03 | 0.71(0.49-1.03) | 0.07 | 0.81(0.55-1.20) | 0.29 |
| 50.0–74.9(nmol/L) | 326 | 3234 | 0.49(0.34-0.72) | <0.001 | 0.55(0.36-0.83) | 0.01 | 0.67(0.43-1.04) | 0.07 |
| >75.0(nmol/L) | 195 | 2525 | 0.40(0.26-0.61) | <0.001 | 0.46(0.29-0.74) | 0.002 | 0.58(0.35-0.96) | 0.03 |
| **Cadmium** |  |  |  |  |  |  |  |  |
| <0.23(ug/l) | 84 | 2127 | 1.00 [Reference] |  | 1.00 [Reference] |  | 1.00[Reference] |  |
| 0.23-0.4(ug/l) | 212 | 2956 | 0.91(0.64-1.29) | 0.58 | 0.92(0.66-1.29) | 0.62 | 0.89(0.64-1.24) | 0.49 |
| 0.4-0.6(ug/l) | 207 | 1695 | 1.02(0.74-1.42) | 0.90 | 1.07(0.77-1.49) | 0.68 | 1.00(0.73-1.36) | 0.99 |
| >0.6(ug/l) | 333 | 2168 | 1.76(1.27-2.45) | 0.001 | 1.73(1.24-2.41) | 0.002 | 1.40(1.01-1.93) | 0.04 |
| **CRP** |  |  |  |  |  |  |  |  |
| <0.09(mg/dL) | 148 | 2075 | 1.00 [Reference] |  | 1.00 [Reference] |  | 1.00 [Reference] |  |
| 0.09-0.25(mg/dL) | 260 | 2445 | 1.06(0.85-1.32) | 0.62 | 1.03(0.84-1.27) | 0.77 | 1.06(0.85-1.34) | 0.59 |
| 0.25-0.58(mg/dL) | 203 | 2218 | 1.07(0.83-1.38) | 0.59 | 1.00(0.77-1.29) | 0.99 | 1.06(0.81-1.39) | 0.64 |
| >0.58(mg/dL) | 225 | 2208 | 1.65(1.29-2.12) | <0.001 | 1.55(1.21-1.99) | 0.001 | 1.60(1.22-2.10) | 0.001 |

^a^ Model 1: adjusted for age

^b^ Model 2: Model 1, additionally adjusted for education, marital status, race, ratio of family income to poverty

^c^ Model 3: Model 2, additionally adjusted for BMI, drinking, smoking, stroke, physical activity

**Table S6 Subgroup analysis for age on the association between serum 25(OH)D，Cadmium , CRP and all-cause mortality among participants with non-diabetes**

| **All-cause mortality（<65）** | **Deaths** | **Total population** | **Model 1 ^a^ Model 2 ^b^  Model 3^c^** | | | | | |
| --- | --- | --- | --- | --- | --- | --- | --- | --- |
|  |  |  | **HR (95%CI)** | **P value** | **HR (95%CI)** | **P value** | **HR (95%CI)** | **P value** |
| **Serum 25(OH)D** |  |  |  |  |  |  |  |  |
| <25.0(nmol/L) | 39 | 592 | 1.00 [Reference] |  | 1.00 [Reference] |  | 1.00[Reference] |  |
| 25.0–49.9(nmol/L) | 228 | 4189 | 0.67(0.45-0.99) | 0.04 | 0.84(0.55-1.27) | 0.40 | 0.90(0.59-1.37) | 0.62 |
| 50.0–74.9(nmol/L) | 234 | 5368 | 0.39(0.26-0.60) | <0.001 | 0.58(0.36-0.92) | 0.02 | 0.66(0.41-1.08) | 0.10 |
| >75.0(nmol/L) | 99 | 3450 | 0.28(0.18-0.44) | <0.001 | 0.45(0.27-0.73) | 0.002 | 0.51(0.30-0.87) | 0.01 |
| **Cadmium** |  |  |  |  |  |  |  |  |
| <0.2 (ug/l) | 37 | 2393 | 1.00 [Reference] |  | 1.00 [Reference] |  | 1.00[Reference] |  |
| 0.2-0.31(ug/l) | 130 | 4434 | 1.31(0.78-2.21) | 0.30 | 1.27(0.76-2.12) | 0.35 | 1.27(0.76-2.10) | 0.36 |
| 0.31-0.6(ug/l) | 162 | 3541 | 1.97(1.13-3.42) | 0.02 | 1.79(1.04-3.07) | 0.04 | 1.64(0.98-2.76) | 0.06 |
| >0.6(ug/l) | 271 | 3231 | 4.25(2.58-6.99) | <0.001 | 3.241.97-5.31) | <0.001 | 2.68(1.59-4.50) | <0.001 |
| **CRP** |  |  |  |  |  |  |  |  |
| <0.07(mg/dL) | 97 | 3064 | 1.00 [Reference] |  | 1.00 [Reference] |  | 1.00 [Reference] |  |
| 0.07-0.19(mg/dL) | 159 | 3834 | 1.28(0.93-1.75) | 0.13 | 1.26(0.93-1.72) | 0.14 | 1.23(0.90-1.68) | 0.19 |
| 0.19-0.45(mg/dL) | 149 | 3311 | 1.46(1.05-2.03) | 0.03 | 1.36(0.96-1.91) | 0.08 | 1.27(0.88-1.82) | 0.20 |
| >0.45(mg/dL) | 195 | 3390 | 2.39(1.72-3.32) | <0.001 | 2.01(1.43-2.81) | <0.001 | 1.80(1.24-2.59) | 0.002 |
| **All-cause mortality（≥65）** |  |  |  |  |  |  |  |  |
| **Serum 25(OH)D** |  |  |  |  |  |  |  |  |
| <25.0(nmol/L) | 49 | 118 | 1.00 [Reference] |  | 1.00 [Reference] |  | 1.00[Reference] |  |
| 25.0–49.9(nmol/L) | 396 | 929 | 0.92(0.60-1.39) | 0.68 | 0.94(0.60-1.46) | 0.77 | 1.07(0.70-1.63) | 0.76 |
| 50.0–74.9(nmol/L) | 576 | 1513 | 0.67(0.42-1.06) | 0.09 | 0.69(0.42-1.14) | 0.14 | 0.81(0.51-1.29) | 0.37 |
| >75.0(nmol/L) | 360 | 1117 | 0.65(0.41-1.04) | 0.07 | 0.68(0.41-1.13) | 0.13 | 0.84(0.52-1.36) | 0.47 |
| **Cadmium** |  |  |  |  |  |  |  |  |
| <0.3(ug/l) | 180 | 722 | 1.00 [Reference] |  | 1.00 [Reference] |  | 1.00[Reference] |  |
| 0.3-0.46(ug/l) | 378 | 1148 | 1.16(0.90-1.49) | 0.24 | 1.15(0.90-1.48) | 0.27 | 1.10(0.86-1.43) | 0.42 |
| 0.46-0.6(ug/l) | 363 | 902 | 1.22(0.96-1.55) | 0.11 | 1.18(0.93-1.50) | 0.16 | 1.09(0.86-1.38) | 0.49 |
| >0.6(ug/l) | 460 | 905 | 2.01(1.59-2.54) | <0.001 | 1.91(1.50-2.43) | <0.001 | 1.57(1.21-2.04) | 0.001 |
| **CRP** |  |  |  |  |  |  |  |  |
| <0.1(mg/dL) | 274 | 845 | 1.00 [Reference] |  | 1.00 [Reference] |  | 1.00 [Reference] |  |
| 0.1-0.22(mg/dL) | 354 | 999 | 1.07(0.90-1.27) | 0.41 | 1.06(0.90-1.25) | 0.47 | 1.06(0.89-1.27) | 0.49 |
| 0.22-0.47(mg/dL) | 359 | 920 | 1.33(1.09-1.63) | 0.01 | 1.32(1.07-1.62) | 0.01 | 1.31(1.06-1.62) | 0.01 |
| >0.47(mg/dL) | 394 | 913 | 1.78(1.43-2.21) | <0.001 | 1.73(1.40-2.15) | <0.001 | 1.72(1.36-2.17) | <0.001 |

^a^ Model 1: adjusted for gender

^b^ Model 2: Model 1, additionally adjusted for education, marital status, race, ratio of family income to poverty

^c^ Model 3: Model 2, additionally adjusted for BMI, drinking, smoking, stroke, physical activity
